# The Effectiveness of the Revised Intermittent Preventive Treatment with Sulphadoxine Pyrimethamine (IPTp-SP) in the Prevention of Malaria among Pregnant Women in Northern Ghana

**DOI:** 10.1155/2020/2325304

**Published:** 2020-11-23

**Authors:** Yaa Nyarko Agyeman, Sam Kofi Newton, Raymond Boadu Annor, Ellis Owusu-Dabo

**Affiliations:** ^1^Department of Population and Reproductive Health, School of Public Health, University for Development Studies, Tamale, Ghana; ^2^Department of Global and International Health, School of Public Health, Kwame Nkrumah University of Science and Technology, Kumasi, Ghana; ^3^Medical laboratory Department, Savelugu Municipal Hospital, Savelugu, Ghana

## Abstract

This study investigated the effectiveness of the World Health Organization (WHO)-revised Intermittent Preventive Treatment using Sulphadoxine Pyrimethamine (IPTp-SP) dosage regimen in the prevention of malaria infections in pregnancy. The study involved a prospective cohort of pregnant women who attended the antenatal clinic in four health facilities (Tamale Teaching Hospital, Tamale West Hospital, Tamale Central Hospital, and Tamale SDA Hospital) within the Tamale metropolis. Data collection spanned a period of 12 months, from September 2016 to August 2017, to help account for seasonality in malaria. The study included 1181 pregnant women who attended antenatal clinics in four hospitals within the metropolis. The registers at the facilities served as a sampling frame, and the respondents were randomly sampled out from the number of pregnant women available during each visit. They were enrolled consecutively as they kept reporting to the facility to receive antenatal care. The participants were stratified into three groups; the no IPTp-SP, <3 doses of IPTp-SP, and ≥3 doses of IPTp-SP. The participants were followed up until 36 weeks of gestation, and blood samples were analyzed to detect the presence of peripheral malaria parasites. At the end of the study, 42.4% of the women had taken at least 3 doses of SP based on the revised WHO IPTp-SP policy. Pregnant women who had taken at least 3 doses of IPTp-SP had a malaria prevalence of 16.9% at 36 weeks of gestation, compared to 35.8% of those who had not taken IPTp-SP. In the multivariable logistic regression, those who had taken ≥3 doses of SP were associated with 56% reduced odds (aOR 0.44, CI 0.27–0.70, *P* = 0.001) of late gestational peripheral malaria, compared with those who did not take SP. IPTp-SP served under three or more doses provided a dose-dependent protection of 56% against maternal peripheral malaria parasitaemia detectable at the later stages of gestation (36 weeks). Since the dose-dependent potency of IPTp-SP depletes with time, there is the need for research into more sustainable approaches that offer longer protection.

## 1. Background

Malaria in pregnancy still remains a public health problem and from 2010, more than 120 million pregnancies in malaria-endemic regions of the world have been suffering yearly malaria-related infant mortality within the range of 75 000–200 000 [1]. In sub-Saharan Africa, *Plasmodium falciparum* is reported to be the main infecting parasite, which is responsible for 99% of all malaria in pregnancy [2]. According to the Ghana Health Service (GHS), malaria is the leading cause of morbidity in Ghana. In 2012, malaria accounted for 16.8% of the hospitalizations of pregnant women and 3.4% of maternal mortalities [3]. In areas where the parasite transmission is stable, 25 million pregnant women are at risk of malaria infections annually [4, 5]. Hence, health planners have to implement operative measures that control malaria infections throughout the period of pregnancy in order to reduce the risk of malaria-induced birth outcomes [6].

IPTp-SP is the main intervention to prevent malaria during pregnancy [7]. In the past, a systematic review of clinical trials had advocated for the intake of two doses in the first and second pregnancies to effectively reduce the likelihood of severe anaemia, malaria in pregnancy, low birth weight, and perinatal mortality [8, 9]. Later evidence indicated that the use of two SP doses had limited prophylaxis potency and effectiveness in protecting pregnant women against malaria parasitaemia, placental malaria, maternal anaemia, and low birth weight (LBW) [10–15]. Furthermore, the prophylaxis duration of the two doses of SP was restricted to four to six weeks of gestation; so, pregnant women remained highly vulnerable to malaria infections during most of their 40 weeks of pregnancy [16]. Due to these findings, the Evidence Review Group (ERG) of the WHO has recommended that at least three SP doses be taken under Directly Observed Therapy (DOT) from the second trimester (first Antenatal Care (ANC) visit) until delivery to help improve maternal and neonatal health outcomes [5].

Ghana initiated the new WHO IPTp-SP recommendation in 2014, and pregnant women are expected to take a minimum of three doses of SP and a maximum of five doses, with each dose taken through directly observed therapy (DOT). The first dose is taken at the first ANC enrolment in the second trimester and the remaining doses are scheduled to be taken at four-week intervals during regular monthly antenatal visits until delivery [17]. The revised policy has been scaled up in all the regions of Ghana including the 26 districts of the Northern Region, but its effectiveness in reducing maternal malaria parasitaemia after implementation remains unknown. There are several published studies on the superiority of 3 doses of SP over 2 doses; however, most of these studies that led to this policy change were conducted in East Africa [9, 12, 18–22].

Few years after the change of the IPTp-SP policy, some studies have questioned the fundamentals of the policy upgrade. For instance, Igboeli et al. [23] in 2018 reported that the review of the malaria chemoprophylaxis policy worsens the malaria burden among pregnant women. The authors reported 13% depreciation of the national usage of IPT services after policy change [23]. Moreover, a 2014 study by Asundep et al. reported that taking a single IPTp-SP dose protected pregnant women better than multiple dosing in Kumasi, Ghana [24]. In a study conducted in Navorongo of Northern Ghana, the authors reported that higher SP doses were not able to protect pregnant women against episodes of malaria [25].

Relatedly, the IPTp-SP programme delivers antimalarial agents to all pregnant women [26]. This potentially creates local variations of malaria endemicity in countries like Ghana. Thus, the effectiveness of IPTp-SP against malaria during pregnancy would depend on the local malaria transmission intensity as well as the degree of the established protective immunity [27]. Therefore, the aim of the study was to assess the effectiveness of the revised Intermittent Preventive Treatment with Sulphadoxine-Pyrimethamine (IPTP-SP) in the prevention of malaria in pregnancy in Northern Ghana.

## 2. Methods and Materials

### 2.1. Study Design

The study involved a prospective cohort of pregnant women who attended antenatal clinic in four health facilities in the Tamale Metropolis (Tamale Teaching Hospital, West Hospital, Central Hospital, and Seventh Day Adventist Hospital). Pregnant mothers that met the study inclusion criteria and sought antenatal care in any of the study facilities were recruited.

The independent variable was IPTp-SP doses and the primary outcome was maternal peripheral malaria parasitaemia. The register at the facilities served as a sampling frame and respondents were randomly sampled out of the number of pregnant women available during each clinic visit. They were enrolled consecutively as they kept reporting to the facility to receive antenatal care.

### 2.2. Study Population

The study population was sampled from pregnant women in the Northern region of Ghana who attended antenatal clinic at any of the selected study sites, namely, Tamale Teaching Hospital, Tamale West Hospital, Tamale Central Hospital, and Tamale Seventh-Day Adventist Hospital. All participants were at 16 weeks of gestation and/or had experienced quickening.

### 2.3. Inclusion Criteria

Pregnant women with at least 16 weeks' gestation and those who attended and delivered in the antenatal clinic were included in the study.

### 2.4. Exclusion Criteria

Pregnant women who migrated out of the study area before the end of the study period; women who did not deliver in any of the study facilities; and women who tested positive for HIV/AIDS, syphilis, hepatitis B, and sickle cell were excluded the study.

### 2.5. Sample Size Estimation

The ANC attendance in 2015 for West hospital, Tamale Teaching hospital, Central, and SDA hospital was 19206, 16293, 22409, and 7000, respectively, totaling 64908. Using an estimated proportion of 85% at 95% confidence level and a precision of 3%, assuming a design effect of 2, the sample size obtained was calculated as 1080. We adjusted for a 10% nonresponse rate and the required sample size was increased to 1188.

The number of pregnant women sampled from each study facility was determined by dividing the respective facility antenatal attendance in 2015 with the total attendance of the year (2015) and multiplying the proportion by the study sample size. A quota of 298, 410, 352, and 128 pregnant women were allocated to Tamale Teaching hospital (TTH), Central, West, and SDA hospital, respectively.

### 2.6. Ethical Consideration

Ethical clearance was obtained from the Committee on Human Research, Publication and Ethics of Kwame Nkrumah University of Science and Technology/Komfo Anokye Teaching Hospital with reference number CHRE/AP/375/16. Written and verbal consent was obtained from all participants.

### 2.7. Data Collection Techniques and Tools

A pre-validated questionnaire was developed using extracts from the Multiple Indicator Cluster Survey (MICS) with an Enhanced Malaria Module and Biomaker (2011) [28] on maternal and new born health, originally designed by UNICEF, and also from the published studies [10, 12, 16, 22, 29, 30]. The study questionnaire was designed to obtain the following variables: sociodemographic information (maternal age, marital status, education, residence, ethnicity, and occupation) and insecticide-treated net (ITN) utilization. The questionnaire also captured the intervention history (SP and ITN usage). The data collection spanned a period of 12 months, from September 2016 to August 2017, to help account for seasonality in malaria. To detect malaria parasitaemia at 36 weeks, blood samples were taken from the participants and processed for microscopic analysis at the Tamale Teaching Hospital Laboratory.

Data extracted from the maternal health record book were cross-checked with the register at the ANC in accordance with the study protocol and the participants were subsequently interviewed directly to corroborate the validity of all obtained information.

### 2.8. Laboratory Testing of Malaria Parasites

Venous blood sample (3 mL) was collected from each participant into an EDTA tube. A drop of blood was placed on a clean frosted microscope slide and was spread to a diameter of 2 cm to make a thick blood smear. All slides were labeled with the corresponding participant identification number used for the study. The blood smears were allowed to air-dry and fixed with absolute ethanol on the field before transporting to the Tamale Teaching Hospital laboratory for reading. At the laboratory, the smears were flooded with 5% Giemsa solution and allowed to stand for 5 minutes. The slides were washed and air-dried before microscopy [31, 32]. All slides were observed under the oil immersion objective lens and blood smears were classified as negative if no malaria parasites were identified after 1,000 white blood cell (WBC) count and positive if malaria parasites were detected at any stage of the WBC count. The microscopy was done by two different senior medical laboratory scientists. All the study slides have been stored in slide storage boxes for five years.

### 2.9. Data Analysis

All data entry and management were conducted using the Microsoft Excel version 16 and exported to STATA 14 for analysis. Categorical variables were compared using Chi-square tests to measure the statistical significance of the calculated proportions. Data on SP intake was grouped into no IPTp-SP doses, one or two (<3) IPTp-SP doses, and greater than or equal to three (≥3) IPTp-SP doses. The results obtained from the three study groups were compared and associations were drawn between the doses of IPTp-SP taken and maternal malaria parasitaemia at 36 weeks of gestation. Binary logistic regression was computed to determine the risk associated with the exposure variable (SP ingestion) and the study outcome (maternal parasitaemia at 36 weeks of gestation). *P* < 0.05 was considered statistically significant.

## 3. Results

### 3.1. Sociodemographic Characteristics of Study Participants

A total of 1188 pregnant women were sampled from four antenatal clinics in the Tamale metropolis. However, only 1181 were used in the final analysis because 7 were lost to follow-up. As shown in Table 1, the majority (34.1%) were below 24 years old, married (92.8%), Dagomba by ethnicity (77.1%), practiced Islam (89.2%), and were urban dwellers (66.6%). Furthermore, approximately half (49.5%) of women had no formal education and engaged in petty trading (39.4%) (Table 1).

### 3.2. Reproductive Health and Obstetric History of Participants

The majority of the study participants had visited the antenatal clinic for the first time during their second trimester of pregnancy (53.9%), were multigravidae (47.6%) and multiparous (45.1%). Approximately 64.7% owned ITNs and 44.3% used them at home. Malaria parasitaemia at registration during the first ANC visit was low (4.5%) among the participants (Table 2).

### 3.3. Prevalence of Malaria in Pregnancy

**Figure 1:**
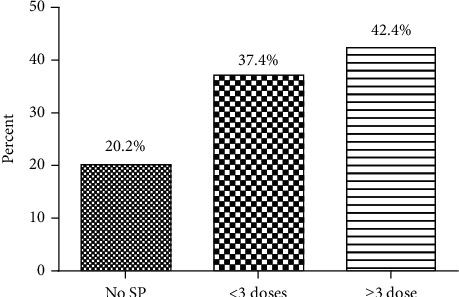
Reported usage of SP among the study respondents.

As shown in Figure 1, 20.2% of the pregnant women reported no usage of IPTp-SP doses, 37.4% reported usage of 1 or 2 doses of IPTp-SP doses, while 42.4% of the women reported adherence to the WHO-recommended IPTp-SP dose policy (at least 3 doses).

The overall prevalence of malaria was 25.9% in the study population. The malaria prevalence was 35.8% in the no SP group, 30.8% among those who took 1 or 2 SP doses, and 16.9% among those who took three or more doses of SP. There was a significant relationship between the reported SP usage and peripheral malaria parasitaemia at 36 weeks of gestation (*X*^2^ = 14.73, *P* < 0.001). Malaria prevalence decreased with increased IPTp-SP use. IPTp-SP showed a dose-dependent association with late gestational malaria parasitaemia in pregnancy (36 weeks) (*P* < 0.001). It could be seen that there was an association between reported ITN/SP use and malaria prevalence (*X*^2^ = 26.75, *P* < 0.001). Pregnant women who neither use ITN nor IPTp-SP had the highest prevalence (38.8%), compared to those who reported combined use of ITN and IPTp-SP (18.4%), only ITN (30.6%), and only IPTp-SP (27.7%). There was not much difference in the prevalence of malaria among pregnant women who reported usage of either ITN (30.6%) or IPTp-SP (27.7%) (Table 3).

### 3.4. Association between IPTp-SP Doses and Malaria Prevalence in Pregnancy

As shown in Table 4, there was a significant association between SP use and the prevalence of malaria. The use of ≥3 doses of IPTp-SP was associated with 56% decrease in the risk of peripheral parasitaemia (aOR 0.44; 95%CI 0.27–0.70;*P* = 0.001 ), compared to those who did not take any IPTp-SP (aOR 0.96; 95%CI 0.63–1.47; *P* = 0.86). There was no evidence that 1 or 2 doses of SP provide protection. From the table, it was seen that those who reported combined use of ITN and IPTp-SP were 48% less likely to get malaria (aOR 0.52; 95%CI 0.31–0.89); (*P* = 0.02), compared to pregnant women who neither used ITN nor IPTp-SP.

## 4. Discussion

### 4.1. Prevalence of Malaria in Pregnancy

The implementation of the IPTp-SP policy had been saddled with frequent shortages of SP, which has denied pregnant women on antenatal appointments access to the programme drug (SP) in many parts of the malaria-endemic regions in sub-Saharan Africa [29]. Furthermore, some women tend to be inconsistent with their visits to the antenatal clinic and this has contributed to inequalities of SP supply [30].

The findings of this study showed that 42.4% of the pregnant women adhered to the WHO-recommended ≥3 IPTp-SP doses. This finding was consistent with similar reports in the Western region of Ghana (47.7%) [30], Burkina Faso (49.2%) [33], and Tanzania (40.6–52.6) [34]. However, our measured percentage of reproted IPTp-SP uptake (42.4%) was higher than published ranges in Uganda (7.0-8.0%) [35], north-western Tanzania (6.0%) [28], and Gambia (3.8%) [33]. This might be due to differences in implementation challenges, procurement bottlenecks, variations in the intensity of malaria transmission geographically, health worker delays, and irregular subscription of the IPTp-SP services by pregnant women [16, 29, 30, 36, 37].

In a high malaria intensity country like Ghana, increased SP dosing (at least three doses) was required to protect pregnant women against gestational malaria [16, 37]. The prevalence of malaria at 36 weeks of gestation was higher in non-SP users (35.8%), compared to pregnant women who used at least one dose of SP users (23.4%). This is in agreement with a previous study in Takoradi (Ghana) by Orish et al. and other published results elsewhere in Africa [16, 29, 30]. In addition, in Tanzania, non-IPTp-SP users recorded higher malaria prevalence in pregnancy (41.7–43.1%) than women who took two (25.3–36.8%) or at least three (14.7–15.4%) SP doses [16, 29]. Again, the prevalence of malaria at 36 weeks of gestation was significantly decreased from 35.8% in non-SP dose users, compared to 16.9% in ≥3 SP dose users. The post IPTp-SP malaria prevalence was inconsistent with published percentages in Benin (4–16%) [37], Nigeria (7.7%) [33], and Ghana (7.9–11.2%) [25, 38]. Antenatal clinics play a significant role in combating maternal and infant mortality. This might be the reason why pregnant women saw the need to take at least three doses of SP.

The prevalence of malaria at 36 weeks of gestation was high among pregnant women who neither used ITN nor IPTp-SP (38.8%), compared to those who combined the use of ITN and IPTp-SP (18.4%). Again, the prevalence of malaria was higher in ITN users (30.6%), compared to those who used IPTp-SP (27.7%). The prevalence seen in the current study was higher than the findings of previous studies in Ghana, Nigeria, and Kenya. The prevalence of malaria was 10.5% among ITN users in the Ashanti region of Ghana [39]; 7.2%, 8.6%, and 4.5% among ITN, IPTp-SP, and both IPTp-SP and ITN users, respectively, in Nigeria [40]; and 12.8% among ITN users in Kenya [41]. The difference in the prevalence could be attributed to changes in geographical locations, level of adherence, and reduced parasite clearance. The utilization of malaria in pregnancy interventions in sub-Saharan Africa was influenced by the individual, the health provider, the system, and/or community factors. These factors affect the intake of IPTp-SP and women often do not take the full doses required to treat infections and provide adequate prophylaxis [42]. The study results confirmed the existence of differences in the protective effect of ITN and IPTp-SP. However, IPTp-SP may still be protective at the individual and community level, unlike in Malawi, where high ITN coverage like that recorded in this setting (>60.0%) masked the effect of IPTp-SP at the community level [43]. The current study findings suggest that the combined use of ITN and IPTp-SP provided additional protection against the prevalence of late gestational malaria (18.4%). In malaria-endemic Africa, combining the usage of IPTp-SP and ITN provides synergistic protection against malaria in pregnancy [43].

### 4.2. Association between IPTP-SP, ITN, and Malaria Prevalence at 36 Weeks of Gestation

IPTp-SP utilization showed dose-dependent protection against the odds of peripheral malaria infections at 36 weeks of gestation. Intake of 1 or 2 doses provided no protection (*P* = 0.86) while higher doses of three or more provided 56% less likelihood (*P* = 0.001) of late gestational (36 weeks) peripheral malaria. Arinaitwe and colleagues also found that 2 doses of SP could no longer treat and provide malaria prophylaxis to pregnant women enrolled in the IPTp-SP programme [10]. This also corresponds with earlier studies in Tanzania by Mosha et al., and Mporogo et al., where high SP dose (≥3) was associated with 60–80% reduced odds of third trimester malaria parasitaemia [16, 29]. The findings of this study also agree with previous studies in Ghana by Orish et al. [30], and within the West African subregion, where the protective efficacy of SP was greater with three or more doses (63–72% reduced likelihood), compared to doses below three (13–36% reduced likelihood) [44]. This reiterates the position of malarial authors that higher SP doses (≥3) provide better protection from malaria during pregnancy [12, 16, 18, 29, 30, 44, 45].

The findings show that the maximum protection against malaria was seen among pregnant women who took 3 or more doses of IPTp-SP, that is, 56% less likelihood of malaria under at least three SP doses. This suggests that the malaria immunity of the study participants remained optimal after three or more IPTp-SP doses. Furthermore, the protective efficacy of SP in our study under three or more doses shifted closer to the lower bracket of protection. That is, 56% versus 60–80% in Tanzania [16, 29], and 56% versus 63–72% in the West African subregion [44]. This might imply that the prevailing factors, such as high parasite transmission intensity, suboptimal use of high SP doses, and the timing of the SP intake, could abruptly shorten the antimalarial protection of at least three SP doses in the study area. Thus, the antimalarial protective potency of three or more doses of IPTp-SP in the study area might be short-lived. This implies that pregnant women could still be in danger of late malaria infection (around 36 weeks) and maternal anaemia during the period where iron and folate are in high demand prior to delivery [46]. In spite of this feat, other studies have reported the loss of efficacy of SP due to the emergence of SP-resistant parasites [43, 47, 48]. Malaria parasites resistant to SP are now spreading and taking dominance because of changing malaria epidemiology [48]. This calls for the need to consider the use of other interventions [49] such as mefloquine as IPT, which was found to be more potent for the prevention of malaria in pregnancy and other adverse effects in pregnancy in a Cochrane Review [50].

Pregnant women who used IPTp-SP, at least one or more times, were not protected in the multivariable regression, compared to those who took three or more doses of IPTp-SP. Using ITN alone without IPTp-SP did not offer maximum protection against malaria. Pregnant women who combined the use of ITN and IPTp-SP were 48% less likely to get malaria, compared to those who used no intervention. These findings are similar to a study conducted in Cameroon [51]. From the study, it could be seen that combining the use of the two interventions (ITN and IPTp-SP) during pregnancy offers the best protection against malaria in pregnancy. This could be attributed to the effectiveness of the SP drug to clear most parasitaemia in the pregnant women and the additional protection provided by bed nets.

### 4.3. Limitations

This study detected maternal peripheral malaria parasitaemia with the aid of a microscope. However, peripheral parasite densities can remain below the levels that can be detected using a microscope (submicroscopic infections), while parasites either inhabit or do not inhabit the placenta (placental malaria) [52]. Again, analysis of placental samples gives a better description of Malaria in pregnancy. This method logically outlines malaria infections that occurred throughout pregnancy and can classify them as infections that occurred before the first SP dose, or during dose intervals, or past infections [16]. Thus, the burden of malaria may have been underestimated in this study [39]. Yet, the microscopy method can detect malaria parasites in the peripheral blood of most pregnant women who have placental malaria infections of different parasite intensities [53]. Moreover, microscopes can detect mild (62%) and moderate (33%) parasitaemia in the peripheral blood better, respectively, than methods that use placental samples (35% for mild and 38% for moderate parasitaemia) [52]. In addition, microscopy concentrates a greater volume of blood to maximize parasite detection and remains the best method for malaria diagnosis in resource-deficient settings like the study context [2].

Although we attempted to control for known confounding variables at the design and analysis stages, it is possible that biases of nonverifiable claims might have influenced our results. For instance, we could not verify the claims of reported use or nonuse of SP and ITN among the participants. The prospective nature of our study design (cohort study) allowed for a verifiable measurement of the use of SP, unlike studies that relied on the records of antenatal cards and interview answers [10]. In spite of this, our study was strengthened by the high usage of IPTp-SP, that is, 79.8% of the pregnant women took at least one dose of SP, with 42.4% of them taking three or more SP doses. This showed that the majority of the participants remained in the study and demonstrated positivity of completing their SP doses. Hence, our study was more robust and the findings might be closer to the true representation of the general population than the values observed elsewhere [54]. Furthermore, our study categorized pregnant women into three groups (no SP use, 1 or 2 SP use, and ≥3 SP use) with comparable usage of SP doses of 20.2%, 37.4%, and 42.4%, respectively. This powered the study to compare the outcomes between groups that took no SP, one to two SP doses, and three or more SP doses.

## 5. Conclusion

Pregnant women in the study area still carried high malaria parasitaemia in their peripheral blood at week 36 of gestation in spite of 42.4% of them complying with the revised dosage of at least three IPTp-SP doses at 36 weeks of gestation. IPTp-SP utilization was associated with dose-dependent protection against the odds of peripheral malaria parasitaemia at 36 weeks of gestation. Thus, some level of protection was achieved among the pregnant women who took at least 3 doses of SP (56%). The results of this study support the position of the WHO revised IPTp-SP policy implemented in Ghana since 2014, which recommends the intake of at least three SP doses. This was shown to be beneficial for decreasing the odds of malaria infection during pregnancy, especially around 36 weeks. Therefore, strategies that maximize the coverage of three or more SP doses should be enhanced in the antenatal system such as taking the IPTp-SP under directly observed therapy (DOT). Furthermore, IPTp-SP use targets could be set for antenatal clinics at the community level; i.e., at least 84% of the pregnant women in the community should complete eight maternity care appointments and take five SP doses annually.

Again, the use of both ITN and IPTp-SP reduced the odds of malaria in pregnancy, compared to using a single intervention. Pregnant women should be educated on the combined usage of ITNs and IPTp-SP during pregnancy to help achieve significant results in the struggle to reduce malaria-related complication in pregnancy.

## Figures and Tables

**Table 1:** Sociodemographic characteristics of study participants.

Sociodemographic variables	Frequency (*n*)	Percent
*Age group (years)*
<24	403	34.1
25–29	383	32.4
30–34	248	21
35+	147	12.5

*Marital status*
Married	1096	92.8
Cohabiting	39	3.3
Widow	2	0.2
Single	44	3.7

*Religion*
Muslim	1053	89.2
Christian	128	10.8

*Residence/locality*
Rural	162	13.7
Peri-urban	232	19.6
Urban	787	66.6

*Educational level*
No school	585	49.5
Primary	253	21.4
Secondary	178	15.1
College/tertiary	165	14.0

*Occupation*
Farmer	44	3.7
Artisan	235	19.9
Salaried employment	149	12.6
Petty trading	465	39.4
Business owners	23	1.9
Food vendor	26	2.2
Domestic activities	10	0.9
Student	56	4.7
Unemployed	170	14.4
Other	3	0.3

*Ethnicity*
Dagomba	910	77.1
Other	271	22.9

Data are presented as count and percent.

**Table 2:** Reproductive health and obstetric history of participants.

Variables	Frequency (*n*)	Percent
*Gravidae*		
Primigravidae	349	29.6
Secundigravidae	269	22.8
Multigravidae	563	47.6

*Parity*		
Nulliparous	391	33.1
Primiparous	257	21.8
Multiparous	533	45.1

*Trimester at 1st ANC*		
1st trimester (0–12 weeks)	507	42.9
2nd trimester (13–24 weeks)	636	53.9
3rd trimester (25 to term)	38	3.2

*ITN ownership*		
Yes	764	64.7

*ITN use*		
Yes	523	44.3

*Frequency ITN use*		
Every evening	230	44
Once a while	293	56

*Malaria parasitaemia at registration*		
Negative	1127	95.5
Positive	54	4.5

Data presented as count and percent.

**Table 3:** Prevalence of malaria at 36 weeks of gestation.

Variable	Malaria parasites at 36 weeks, *n* (%)		*X* ^2^	*P*-value
	Negative, 849 (74.1%)	Positive, 297 (25.9%)		
*Reported usage of SP*			14.73	<**0.0001**
Did not take SP	149 (64.2)	83 (35.8)
Took SP	700 (76.6)	214 (23.4)

*SP dosage*			37.86	<**0.0001**
No SP	149 (64.2)	83 (35.8)
<3 dose	296 (69.2)	132 (30.8)
≥3 dose	404 (83.1)	82 (16.9)

*Reported ITN/SP usage*			26.75	**<0.001**
No ITN/SP use	90 (61.2)	57 (38.8)
Only SP use	355 (72.3)	136 (27.7)
Only ITN use	59 (69.4)	26 (30.6)
Both SP and ITN use	345 (81.6)	78 (18.4)

Data are presented as count (percent). Categorical variables are compared using the Chi-square test, and *P* < 0.05 are considered statistically significant.

**Table 4:** Multivariable analysis between IPTp-SP and maternal parasitaemia at 36 weeks of gestation.

Variable	Univariate regression		Multivariable regression	
	cOR 95% CI	*P*-value	aOR (95% CI)	*P*-value
*Parasitaemia at 36 weeks*				
No SP	1.00 (ref)		1.00 (ref)	
<3 doses	0.86 (0.57–1.12)	0.20	0.96(0.63–1.47)	0.86
≥3 doses	0.36 (0.25–0.52)	**<0.001**	0.44(0.27–0.70)	**0.001**

*Reported ITN/SP usage*				
No SP/ITN use	1.00 (ref)		1.00 (ref)	
Only SP	0.60 (0.41–0.89)	**0.01**	0.82 (0.50–1.37)	0.46
Only ITN	0.70 (0.39–1.23)	0.21	0.96 (0.46–2.00)	0.92
Both SP and ITN	0.36 (0.24–0.54)	**<0.001**	0.52 (0.31–0.89)	**0.02**

cOR = crude odd ratio; aOR = adjusted odd ratio; 95% CI = 95% confident inferential; *P* < 0.05 are considered statistically significant; ref = reference (1.00).

## Data Availability

The datasets used during the current study are available from the corresponding author on reasonable request.
